# The Quality Mental Health Care Network: A roadmap to improving quality mental healthcare in Canada

**DOI:** 10.1177/0840470420974713

**Published:** 2021-01-08

**Authors:** Emily J. Follwell, Siri Chunduri, Claire Samuelson-Kiraly, Nicholas Watters, Jonathan I. Mitchell

**Affiliations:** 1 153026 HealthCareCAN, Ottawa, Ontario, Canada.; 2 434957 Mental Health Commission of Canada, Ottawa, Ontario, Canada.

## Abstract

Although there are numerous quality of care frameworks, little attention has been given to the essential concepts that encompass quality mental healthcare. HealthCare*CAN* and the Mental Health Commission of Canada co-lead the Quality Mental Health Care Network (QMHCN), which has developed a quality mental healthcare framework, building on existing provincial, national, and international frameworks. HealthCare*CAN* conducted an environmental scan, key informant interviews, and focus groups with individuals with lived experiences to develop the framework. This article outlines the findings from this scan, interviews and focus groups.

## Introduction

One in five Canadians suffer from a mental illness every year and will need access to quality mental healthcare.^1^ This issue is all the more pressing now with the World Health Organization sounding the alarm that the insidious effects of stress, grief, and anxiety related to the COVID-19 pandemic will most certainly lead to greater incidence of mental distress in the general population and particularly in healthcare workers.^2^ Mental healthcare in Canada is delivered in community or clinical settings through a wide range of health professionals such as family doctors, psychiatrists, psychologists, nurses, and social workers among others. A patchwork of coverage includes provincial and territorial healthcare plans and employer-sponsored health insurance. Individuals seeking mental health services may have to pay out-of-pocket for some professionals. In addition to these factors, access to mental healthcare also differs from province to province or territory and within provincial or territorial borders. The complex nature of mental illness can be exacerbated by the social determinants of health and care can also be delayed because of the stigma and discrimination associated with mental illness, substance use and addiction, and the risk of suicide.

HealthCare*CAN* ^3^ and the Mental Health Commission of Canada (MHCC)^4^ work in partnership to advance the psychological health and safety of Canadian healthcare workplaces and promote quality mental healthcare. In 2013, Canada became the first country in the world to have a National Standard of Canada for Psychological Health and Safety in the Workplace (the Standard).^5^ In 2016, HealthCare*CAN* and MHCC co-created the *By Health, For Health Collaborative* (the Collaborative) and recruited healthcare leaders from over 30 diverse healthcare organizations across Canada to champion the shift in healthcare to focus on mental health in the workplace. The Collaborative’s impact was instrumental in having the implementation of the Standard within healthcare organizations recognized as a leading practice by Health Standards Organization/Accreditation Canada.^6^

At the end of the Collaborative’s 2-year mandate, HealthCare*CAN* and MHCC together created the Quality Mental Health Care Network (QMHCN) to continue building on the successes of the Collaborative. The QMHCN is a pan-Canadian network of health sector leaders dedicated to addressing structural stigma, promoting recovery-oriented practice, and furthering the work of the Collaborative regarding psychologically healthy workplaces. A major initiative of the QMHCN is to lead the development of a framework for quality mental healthcare in the Canadian healthcare context.

## Methods

From an international perspective, the Mental Health Commission of Canada provided insights from their participation in the International Initiative for Mental Health Leadership (IIMHL),^7^ a collaborative between Australia, Canada, England, the Netherlands, the Republic of Ireland, New Zealand, Scotland, Sweden, and the United States, which focuses on mental health and addiction services. The materials from the IIMHL knowledge exchange forum were reviewed extensively as background materials to gain a greater understanding of the best practices and recovery-oriented practices in the international mental healthcare space.

To further inform the development of the framework for quality mental healthcare, HealthCare*CAN* conducted an environmental scan of relevant materials, followed by key informant interviews, and focus groups with individuals with lived experiences.

### Environmental scan

The first step was an environmental scan of existing provincial/territorial, national, and international quality care frameworks. Materials were gathered by conducting on-line searches using keywords and phrases including: “Quality mental health frameworks”; “Quality health frameworks”; “Quality health” + Province/Territory, “Quality mental health” + Province/Territory, and “Hospital quality framework Canada.” A more targeted search of the Health Standards Organization’s web site and Canadian hospital websites for quality improvement initiatives was also conducted.^8,9^ In addition, searches included the academic literature on quality mental health.^10^ Materials were further analyzed for definitions and dimensions of quality healthcare. The initial search yielded nine key results, comprised of seven frameworks and two journal articles. An additional seven frameworks were added through recommendations from network members and experts for a total of 14 quality care frameworks that were analyzed in further detail.

### Key informant interviews

#### Phase I

Purposive sampling was used to identify 24 key informants that met the following criteria:

1. Health professionals with experience in mental healthcare, and
2. Individuals from across the country to provide a broad, pan-Canadian perspective.

Invitations were sent to (1) members of the QMHCN, (2) HealthCare*CAN* member Vice-Presidents of Health Research with expertise in mental healthcare and research, and (3) subject matter experts in framework development, quality improvement, and mental health. In some cases, multiple experts working at the same organization were invited. Twenty informants were available for interviews and represented mental healthcare organizations as well as national healthcare organizations. As part of the QMHCN, members of the MHCC were among the interviewees and were solicited for their participation in the IIMHL and providing an international perspective in addition to their knowledge of the Canadian mental health sector. A total of 13 structured interviews with 20 key informants were conducted between November 4, 2019, and January 23, 2020. The interviews were guided by four questions:

1. What existing quality care framework(s) would be helpful in developing a framework for quality mental healthcare?
2. What quality dimensions common to these frameworks would apply to mental healthcare?
3. What aspects unique to mental healthcare should be incorporated into a framework for quality mental healthcare?
4. What words and/or concepts come to mind when you think of quality mental healthcare?

Upon completion of the interviews, the insights were used to develop a first draft of the framework. An additional seven frameworks were added for further consideration via recommendations of the key informant interviews. The draft was then shared with four subject matter experts. Three of the experts were members of the QMHCN, who also participated in the key informant interviews. The four subject matter experts were consulted to gather feedback on framework development and develop consensus.

#### Phase II

A second phase of consultations was conducted with People with Lived Experiences (PWLE). Virtual focus groups over Microsoft Teams lasting 1 hour each were held with three PWLE committees affiliated with QMHCN member organizations: the MHCC Youth Council, the MHCC Hallway Group, and the Canadian Patient Safety Institute’s (CPSI) partner organization Patients for Patient Safety Canada (PFPSC). The focus groups were initially planned for April 2020 and would have been face-to-face as was the norm before the COVID-19 pandemic but had to be postponed and conducted virtually. Based on the previous interactions of the facilitators from MHCC and CPSI with the PWLE groups, these changes in timing and format did not have a noticeable impact on the attendance or level of engagement of participants.

All three PWLE groups were comprised of volunteers of diverse ages, religions, and races, and Indigenous peoples were represented. The focus groups were held in July 2020 with a total of 29 focus group participants—9 from the Youth Council, 7 from the Hallway Group, and 13 individuals from PFPSC. An abridged version of the Framework was shared with each PWLE committee prior to the focus groups. The following questions guided the discussion:

1. What words and/or concepts come to mind when you think of quality mental healthcare?
2. What aspects unique to mental healthcare should be incorporated into a framework for quality mental healthcare?
3. What gaps, if any, need to be addressed in the framework? Are there dimensions in the framework that should be reworked or eliminated?

Materials were shared with the full patient committee groups and all feedback was presented a second time to the groups to ensure full participation and perspectives of all members. This also ensured that the perspectives of those who were unable to participate in the first round of interviews were not markedly different from those who did participate.

### Revisions and consultations

The draft of the Framework was updated with the PWLE recommendations. As part of an effort to expand and diversify the membership of the QMHCN in its second year, additional members including some of the participants of the focus groups were added to the Network. The revised draft was recirculated among the Network in September 2020 for further input and feedback.

#### Findings from existing frameworks

The 14 quality care frameworks offered valuable insights into the development of a framework for quality mental healthcare. The quality dimensions identified in each framework were found to largely overlap, with accessibility, safety, effectiveness, a healthy workplace culture, and patient-centredness appearing in most frameworks. The Institute for Healthcare Improvement’s Framework for Safe, Reliable, and Effective Care^11^ and Health Standards Organization/CPSI’s Canadian Quality and Patient Safety (CQPS) Framework for Health and Social Services^12^ were examined in greater detail as they were highlighted repeatedly by the key informant interviewees.

The IHI Framework for Safe, Reliable, and Effective Care provides clarity and direction to healthcare organizations on the key strategic, clinical, and operational components involved in achieving safe and reliable operational excellence—a “system of safety,” not simply a collection of stand-alone safety improvement projects. The framework has two foundational dimensions—culture and the learning system—and nine interrelated components: leadership, psychological safety, accountability, teamwork and communication, negotiation, transparency, continuous learning, reliability, and improvement and measurement. IHI’s Quadruple Aim framework was also considered in greater detail.^13^ The Triple Aim framework is structured around three dimensions: better care for individuals, better health for populations, and lower per capita costs. The Quadruple Aim adds a fourth dimension: improving the work life of healthcare providers.

The HSO/CPSI CQPS Framework for Health and Social Services has five dimensions outlined as goals that each play a role in providing safe, high-quality healthcare. The dimensions include (1) people-centred care, (2) safe care, (3) accessible care, (4) appropriate care, and (5) integrated care. The CQPS framework is meant to guide Boards of Directors, healthcare teams, patients, policy-makers, leaders, and the public through Action Guides which outline clear interventions stakeholders can apply to meet the goals of the framework and improve the care provided in all healthcare settings.

Of the international frameworks that were included in the analysis, the Organisation for Economic Co-operation and Development framework views the drivers of high-quality care as the cornerstone of quality improvement with three dimensions: effective, safe, and patient-centered.^14^ The Institute of Medicine meanwhile places importance on high quality care as essential for ensuring desired health outcomes in relation to current professional knowledge and includes six dimensions: effectiveness, efficiency, equity, patient centeredness, safety, and timeliness.^15^

The provincial frameworks (BC Patient Safety and Quality Council,^16^ Health Quality Council of Alberta,^17^ Government of Manitoba,^18^ Health Quality Ontario,^19^ New Brunswick Health Quality Council,^20^ Health Systems of Nova Scotia^21^) had many areas of overlap in quality dimensions such as safe, appropriate, and effective. Only the framework for Nova Scotia emphasizes a healthy workplace environment, while the Manitoba Patient Safety Framework views health quality through a patient safety improvement lens.

#### Findings from key informant interviews

The first question in the guided interviews asked the interviewees to suggest existing quality frameworks which would be helpful in developing a framework for quality mental healthcare. The IHI and HSO/CPSI Frameworks were identified because they are evidence-based, well-known in Canada, and recognize the mental health of healthcare workers. In addition, several participants highlighted IHI’s Quadruple Aim framework.

The interviewees identified people/patient/population-centredness as the most important quality dimension common to the reviewed frameworks. Other dimensions that interviewees felt would apply to mental healthcare included equity, safety and psychological safety, evidence-based, appropriateness, continuity of services, seamless care, accessibility and timeliness, continuous learning and improvement, comprehensiveness, work life, workplace culture, and cultural safety. For the quality dimension of seamless care, they referenced similar terms such as integrated care and coordinated care. These quality dimensions support the concept that the framework should integrate provider and patient perspectives.

The respondents provided a wealth of information about the aspects unique to mental health which should be incorporated into a framework for quality mental healthcare. There are many perspectives to consider as mental healthcare is the opposite of a “one-size fits all” approach. Interviewees noted that mental illness is complex, and patients often have multiple comorbidities, which could include mental or physical health conditions. Stigma can delay early intervention to care, which is integral to mental health and the path to recovery-oriented care.

Many interviewees noted that quality mental healthcare should be patient- and family-focused, integrated across the continuum of care, and provide a seamless transition between inpatient care and the community setting. An interviewee stated that quality mental healthcare is achieved when an individual is living their life with the greatest normalcy that can be retained with the appropriate support or treatment. Quality care should always be the gold standard and must be flexible, evidence-informed, innovative, integrated, compassionate, and stigma-free.

The information gathered from the key informant interviews helped to frame the quality dimensions that are essential from a provider’s perspective. The final framework includes the concepts of continuous learning and improvement and work-life environment to better account for the psychological health and safety of healthcare workers.

#### Findings from PWLE focus groups

The three focus groups provided valuable information regarding the concepts of “quality,” defining it as “quality of life which is meaningful to the individual and their support system.” Quality mental healthcare therefore supports a meaningful quality of life, and as participants articulated, should be individualized, inclusive and equitable, respectful, comprehensive, integrated, effective, evidence-based, “meets people where they are at,” is accountable, and culturally appropriate, safe, and sensitive. Another theme that emerged from all three focus groups was the importance of considering the social determinants of health when addressing mental healthcare and designing a healthcare framework.

Regarding evidence-based care, focus group participants raised the question of who the evidence is supposed to serve and what it means to stakeholders (e.g., patients, providers, caregivers, communities, and policy-makers). They felt that there was a bias towards evidence-based therapies, which may not be effective or address individual needs (e.g., Cognitive Behavioural Therapy), while other non-medical interventions (e.g., peer-support) receive praise from many users yet remain at the bottom of treatment options. In addition, evidence-based therapies are reliant on clinical trial results and there are populations which have been historically excluded from clinical trials. Nonetheless, some reported that evidence-based care needs to extend beyond how mental services are traditionally delivered.

Participants stressed that in the current context of the COVID-19 pandemic, expanding virtual options should be a priority. Specifically, some participants felt that multiple ways of receiving mental health services through phone apps, messaging services, and other digital platforms addressed both the accessible dimension by providing services beyond traditional office hours and the stigma dimension by offering a more neutral space. They also felt that virtual options may increase usage of resources by younger patients, who may otherwise find transportation and financial barriers prohibitive and find texting or communicating on-line consistent with the way they communicate regularly. Participants agreed that the pandemic has led to an expansion of virtual mental healthcare services, and exploring this area is of great interest. While this was out of scope for this Framework, this may serve as the basis for subsequent research.

Self-determination was an important concept for PWLE. An individual receiving care must be able to have input into decisions and be presented with adequate knowledge about the care they are receiving, which allows them to make informed decisions. In their words: “Quality mental healthcare should equip patients with information about their rights, how to navigate the system, and engage the family and caregivers in a respectful fashion addressing their need-to-know questions and concerns regarding their loved one’s treatment, as appropriate.”

Participants reported that the mental healthcare system is by design not accessible. It penalizes patients for their illnesses and for seeking help. Participants expressed that a welcoming environment and basic customer service every time they access the mental health system is critical. Patients want to feel “cared about” instead of “cared for,” and compassion is very important. However, focus group members felt that the accessibility dimension should mean more than simple healthcare services. It should encompass access to services which address the social determinants of health, including housing, social services, and income support.

Most participants noted that no quality mental healthcare framework could be complete without the inclusion of trauma-informed care. Trauma-informed care recognizes the impacts of trauma and violence on individuals receiving mental health care services. Furthermore, focus group participants raised the concept of cultural competency and pointed out that culturally competent, culturally safe, and culturally appropriate were all unique concepts that should be given proper consideration. People think about mental health differently around the world, and it influences how patients, families, and providers communicate about mental health. Leaders must ensure that care is adequately adapted to different cultural contexts.

People with lived experiences noted that stigma-free might be an aspirational goal. Their recommendation was that inclusivity deserves its own recognition and should be considered separately. Seamless received similar comments and was deemed impossible to achieve. A participant added that seamlessness was unachievable if community-based programs remained underfunded. Focus group participants also recommended that the Framework includes the potential role of upstream preventative mental health supports, such as developing mental health literacy and awareness, for example, through public education in schools.

The comments from the PWLE were integrated into the definition of quality mental healthcare as well as used to rework the quality dimensions. The quality dimension “stigma-free” was revised to “stigma-free and inclusive” to broaden the dimension and address removing various barriers to seeking mental health services. The quality dimension “seamless” was changed to “integrated” to better reflect the necessity of more community-based programs. “Trauma-informed” was identified as a uniquely mental health-related concept by PWLE and was therefore added as an additional dimension to the final framework.

## The resulting framework

### Defining mental healthcare

The frameworks analyzed in the environmental scan and information gathered from the key informant interviews and the focus groups with the PWLE helped to identify several key components of a quality mental healthcare framework. Quality mental healthcare is people-centred, trauma-informed, stigma-free and inclusive, accessible, safe, appropriate, recovery-oriented, integrated, promotes continuous learning and improvement, and ensures that healthcare providers have a safe and comfortable workplace environment.

### Quality dimensions

The quality mental healthcare framework builds on the HSO/CPSI CQPS Framework for Health and Social Services. The IHI Framework for Safe, Reliable, and Effective Care and Quadruple Aim approach to the inclusion of providers in quality care has been carefully considered as well. The framework (Table 1) builds on the five goals of the HSO/CPSI framework (people-centre, safe, accessible, appropriate, integrated) and, based on our findings, adds the following dimensions for the mental healthcare perspective:

- Stigma-free and inclusive
- Trauma-informed
- Recovery-oriented
- Continuous learning and improvement
- Work-life environment

**Table 1.** Framework dimensions of quality mental healthcare

Dimension	Description
Accessible	Having timely and equitable care across the continuum. Promotes prevention and early intervention. Community-based interventions are available.
Appropriate	Care is evidence-informed and culturally competent.
Continuous learning and improvement	Knowledge sharing and capacity building among members of the healthcare workforce. Innovative care is encouraged and supported.
Integrated	Care is continuous across the continuum. Transition into community settings is smooth. Family and/or patient’s support system is involved. Integration with services that address social determinants of health.
People-centred	Care is focused and organized around the health needs and expectations of people and communities rather than on diseases.^6^
Recovery-oriented	Living a satisfying, hopeful, and meaningful life, even when there may be ongoing limitations related to mental health problems and illnesses.^22^
Safe	Keeping people and providers safe from preventable harm. Care is culturally safe for individuals and marginalized populations.
Stigma-free and inclusive	Care addresses drivers of mental health stigma and prevents stigma practices in mental healthcare. Health providers are comfortable in coming forward with their mental health problems and illnesses at work. Addresses multiple layers of stigma (individual, interpersonal, intersectoral, and structural). A need to better support individuals who have experienced stigma and discrimination. Individuals feel respected and valued.
Trauma-informed	Recognizes the impacts of trauma and violence on individuals receiving mental healthcare services.
Work-life environment	A healthy workplace environment supports provider wellness and promotes psychological safety.

Key informant interviewees, subject matter experts, and PWLE identified five dimensions as particularly relevant when addressing quality mental healthcare. *Stigma-free and inclusive*, in addition to addressing all the various levels of stigma that need to be confronted, includes the concept of self-determination of patients, awareness of their rights, and how to navigate the system. Inclusivity is vital to feeling respected and valued and that means being included in one’s own treatment and recovery plans. *Trauma-informed* emphasizes the importance of providers being sensitive to the effects of trauma on people seeking mental health services, being able to recognize signs of trauma, responding appropriately, and avoiding re-traumatization. *Recovery-oriented* includes accommodating the other supports that are needed to ensure a satisfying, hopeful, and meaningful life such as housing, social services, and income supports, etc. Providers should have opportunities for *continuous learning and improvement* by being open to alternative therapies and innovative approaches, while seeking to gather knowledge from experts and employ best practices. To properly address the provider component, the framework must value provider mental health and wellness and optimal *work-life balance*. A mentally unwell provider or a healthcare worker suffering from burnout may have difficulty delivering quality patient care; thus, prevention and promotion of a healthy workplace culture supports quality mental healthcare.

## Leading in quality mental healthcare: Next steps

Based on the unique perspectives of health leaders, PWLE, and the power of the QMHCN, HealthCare*CAN* and the MHCC will prioritize actions to implement our findings into improved practice for mental healthcare services in Canada. While indicators and outcome measures have been considered to operationalize the Framework, guidance from the experts in the field that we interviewed have pointed to the challenges in securing consensus and buy-in from healthcare leaders across the continuum of care and the necessity to keep to a simple framework that connects to broader and accepted Canadian quality frameworks. However, this provides another possible avenue of further development.

The health leaders who we interviewed and who are part of the QMHCN stressed the importance of knowledge translation tools to highlight findings that will be key in advancing improved practice. The development of these resources is currently underway with the support of the QMHCN. Knowledge products will be created including infographics and policy briefs will be used in dissemination of key findings for improving mental healthcare, through HealthCare*CAN*’s and the MHCC’s networks, and social media. HealthCare*CAN* and MHCC will also leverage the support of the QMHCN and the members’ affiliated organizations to spread awareness of the Framework. This includes further collaboration on next steps for the HSO/CPSI CQPS Framework for Health and Social Services in an effort to customize for a mental health environment based on the insights gleaned from our key informant interviews and focus groups.

A comprehensive approach to mental health means that healthcare providers and health leaders must provide quality care that meets the needs of their communities and continuously improve the care provided in all healthcare settings. Our collaborative work has highlighted specific opportunities for improvement in mental healthcare. Solutions must address comprehensive mental healthcare for patients while also considering the mental healthcare needs of the providers. Ultimately, what quality mental healthcare means is “the right mental healthcare, at the right time, by the right team, in the right place.”
